# The Potential of Parsley Polyphenols and Their Antioxidant Capacity to Help in the Treatment of Depression and Anxiety: An In Vivo Subacute Study

**DOI:** 10.3390/molecules26072009

**Published:** 2021-04-01

**Authors:** Imane Es-safi, Hamza Mechchate, Amal Amaghnouje, Omkulthom Mohamed Al Kamaly, Fatima Zahra Jawhari, Hamada Imtara, Andriy Grafov, Dalila Bousta

**Affiliations:** 1 Laboratory of Biotechnology, Environment, Agri-Food and Health (LBEAS), Faculty of Sciences Dhar El Mahraz, Sidi Mohamed Ben Abdellah University (USMBA), Fez 30050, Morocco; Imane.essafi1@usmba.ac.ma (I.E.-s.); Amal.amaghnouje@usmba.ac.ma (A.A.); jawhari.fatimazahra@gmail.com (F.Z.J.); Dalila.bousta@usmba.ac.ma (D.B.); 2 Department of Pharmaceutical Sciences, College of Pharmacy, Princess Nourah Bint Abdulrahman University, Riyadh 11564, Saudi Arabia; omalkmali@pnu.edu.sa; 3 Faculty of Arts and Sciences, Arab American University Palestine, Jenin 240, Palestine; hamada.tarayrah@gmail.com; 4 Department of Chemistry, University of Helsinki, 00100 Helsinki, Finland; Grafov.andriy@helsinki.fi

**Keywords:** *Petroselinum sativum*, polyphenol, anxiolytic, antidepressant-like, antioxidant, pharmacology, herbal medicine, natural products

## Abstract

Depression and anxiety are major mental health problems in all parts of the world. These illnesses are associated with a number of risk factors, including oxidative stress. Psychotropic drugs of a chemical nature have demonstrated several side effects that elevated the impact of those illnesses. Faced with this situation, natural products appear to be a promising alternative. The aim of this study was to evaluate the anxiolytic and antidepressant effects of the *Petroselinum sativum* polyphenols in vivo, as well as its correlated antioxidant properties in vitro. Anxiolytic activity of the extract (50 and 100 mg/kg) was evaluated using the open field and the light-dark chamber tests, while the antidepressant activity was evaluated using the forced swimming test. The antioxidant activity of the extract was evaluated by the 2,2-diphenyl-1-picrylhydrazyl (DPPH) free radical test and the FRAP (iron-reducing capacity) test. The phenolic extract showed very powerful anxiolytic and antidepressant-like effects, especially at a dose of 100 mg/kg, decreasing the depressive behavior in mice (decreased immobility time) and also the anxiolytic behavior (tendency for discovery in the center and illuminated areas) better even than those of paroxetine and bromazepam (classic drugs) concomitant with those results the extract also showed an important antioxidant capacity. These preliminary results suggest that *Petroselinum sativum* exhibits anxiolytic and antidepressant potential for use as a complement or independent phytomedicine to treat depression and anxiety.

## 1. Introduction

Depression and anxiety disorders have long been considered mental health issues, and they constitute a heavy burden for any society in which stress is omnipresent. The World Health Organization (WHO) predicts that by 2030, depression, as a potentially fatal disease, will be the second leading cause of disability worldwide [1]. According to the latest WHO estimates, more than 300 million people worldwide suffer from depression, of which more than 260 million also have anxiety disorders [2].

The Diagnostic and Statistical Manual of Mental Disorders (DSM-V) defined major depressive disorder as not just an occasional sadness or bout of depression, but rather a depressed mood or loss of interest or pleasure for at least two consecutive weeks, episodes of which are often recurrent [3]. The WHO also defines depression as “a variety of psychological and biological symptoms, such as a general feeling of sadness, loss of pleasure and interest, the feeling of guilt, disturbed sleep (insomnia or drowsiness) and appetite, feelings of fatigue (psychomotor retardation), and lack of concentration”. In the most serious cases, depression can even lead to suicide [4].

Anxiety, as defined by the WHO, is “a feeling of undetermined imminent danger accompanied by a state of malaise, agitation, helplessness, or even annihilation”. It can also be described as a state of anticipatory fear caused by potential and uncertain danger, although its cause is not necessarily conscious, as opposed to fear that arises in the face of real danger. However, these two emotions are characterized by similar physiological (increased blood pressure, sweating, tachycardia, etc.) and behavioral (increased alertness and avoidance) responses [5].

Depression and anxiety are generally treated with psychotropic medications, including antidepressants and anxiolytics (tranquilizers), often chemical in nature, such as paroxetine and benzodiazepines. However, the use of these medications exhibits certain limits, and they may cause unwanted side effects. Bromazepam is prescribed for the treatment of anxiety. However, its consumption comes with a number of negative side effects, including drowsiness, sedation, and memory loss [6]. Difficulty sleeping, loss of appetite, drowsiness, and sexual dysfunction are also typical side effects of paroxetine, an antidepressant from the serotonin reuptake inhibitor (SSRI) family used to manage major depressive disorders [7]. As a result of this limited efficacy, the exploration of new, more effective, and non-toxic therapeutic means is highly appreciated.

Medicinal plants can be an adequate source of new, safe, and effective therapeutics, especially as many are known to exhibit fewer side effects [8]. Multiple medicinal plants have been tested in the above disorders, and they yielded very pleasant results that encouraged further studies to discover antidepressant-like and anxiolytic proprieties of additional plants [9].

*Petroselinum sativum* Hoffm., commonly known as “parsley” (“maadnous” in Arabic), is a member of the Apiaceae family. *Apium crispum* Mill, *Apium petroselinum* L., *Petroselinum hortense* Hoffm and *Petroselinum crispum* (Mill) Fuss. are also synonyms for *Petroselinum sativum* Hoffm. [10]. Parsley, as a culinary herb that originated from the Mediterranean region, has become a globally common herb in modern times [11]. *P. sativum* has a range of beneficial properties, including antioxidant, analgesic, spasmolytic, antidiabetic, immuno-modulating, and gastrointestinal effects [12]. These various benefits may be attributed to the plant’s core components, such as polyphenols (apigenin, quercetin, luteolin, and kaempferol), vitamins, carotenoids, coumarin, and tannins [13]. The Apiaceae family encompasses multiple plants known for their antidepressant and anxiolytic activities like *Coriandrum sativum* L. [14], *Pimpinella anisum* L. [15], *Carum carvi* L. [16] for a concentration ranging between 50 and 200 mg/kg.

In this study, potential antidepressant-like and anxiolytic activities of parsley polyphenols were evaluated for the first time, along with its antioxidant activity, to determine whether there was a correlation.

## 2. Results

### 2.1. Evaluation of the Antioxidant Activity

#### 2.1.1. DPPH Test

**Figure 1:**
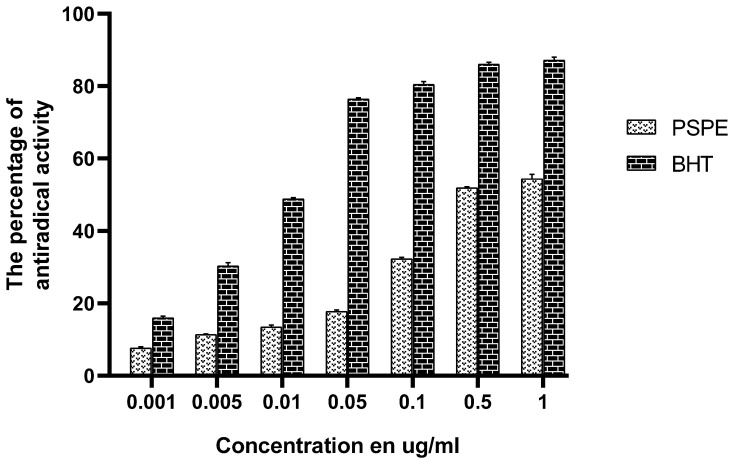
Antioxidant activity of PSPE during the DPPH test. PSPE: Polyphenolic fraction of *P. sativum*, BHT: Butylated hydroxytoluene.

Figure 1 shows the percentage of antioxidant activity as a function of different levels of PSPE and BHTs. The results obtained reveal that our extract and BHT display concentration-dependent antiradical activity. That is to say, the percentage of inhibition of the DPPH radical increases with the concentration of the phenolic extract of *P. sativum* and BHT.

BHT showed the highest activity compared to our extract. For 0.5 μg/mL, BHT reached a maximum inhibition percentage of 90%, which remained constant for a concentration of 1 μg/mL. At the same concentration, the phenolic extract produced a maximum inhibition of 58%.

The synthetic antioxidant BHT showed a very powerful antiradical activity, with an IC_50_ of about 0.024 μg/mL, higher than that recorded for the phenolic extract of *P. sativum* (about 0.184 μg/mL).

#### 2.1.2. FRAP Test

**Figure 2:**
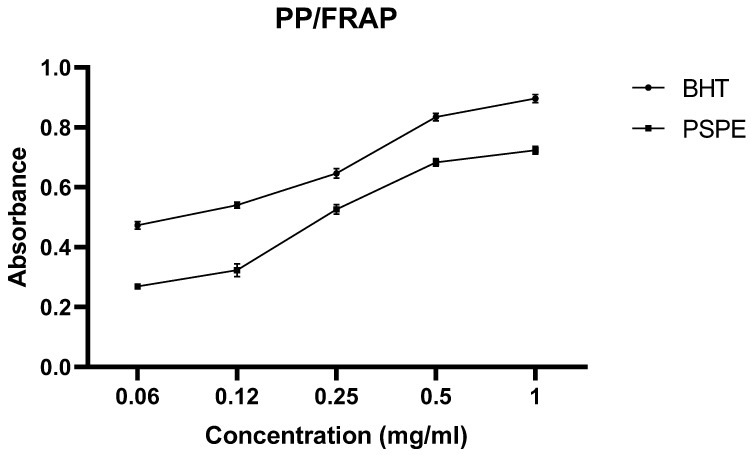
Antioxidant activity of PSPE during the FRAP test. PSPE: Polyphenolic fraction of *P. sativum*, BHT: Butylated hydroxytoluene.

Figure 2 shows the variation in optical density (OD) as a function of the different concentrations of PSPE and BHT (positive control). It can be seen that the percentages of reduction are proportional to the concentration of both the extract and BHT. The latter showed a higher percentage of reduction compared to the extract.

In order to compare the efficiency of the polyphenolic fraction of *P. sativum* (PSPE) with that of BHT, we determined the concentration that reduced 50% of the FRAP (IC_50_).

BHT (The synthetic antioxidant BHT) showed highly potent antioxidant activity with an IC_50_ of about 0.09 μg/mL, higher than that recorded for the phenolic extract of *P. sativum* (about 0.38 μg/mL).

### 2.2. Evaluation of Antidepressant Activity

#### Forced Swimming Test

**Figure 3:**
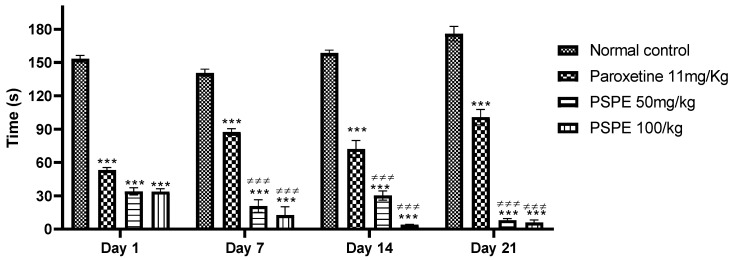
Variation in immobility time during three weeks of treatment in control and treated mice (*** *p* ≤ 0.001 in comparison to negative controls, ≠≠≠ *p* ≤ 0.001 in comparison to positive controls). PSPE: Polyphenolic fraction of *P. sativum*.

The variation in the downtime in the forced swimming test during the three weeks of the experiment is shown in Figure 3. The immobility time during the test was significantly shorter in PSPE-treated mice (PSPE 50 mg/kg (34 s ± 3.286), PSPE 100 mg/kg (33.8 s ± 2.653)) compared to controls (Paroxetine (100.8 s ± 6.837), Vehicle (176 s ± 6.550)).

These results show that the antidepressant effect of the phenolic extract of *P. sativum* is greater than that of paroxetine.

### 2.3. Evaluation of the Anxiolytic Activity

#### 2.3.1. Anxious Behavior in the Open Field

**Figure 4:**
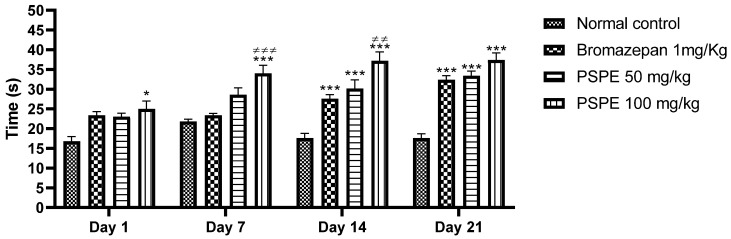
Variation in time spent at the center of the open field during the four-week treatment in control and extract-treated mice (* *p* ≤ 0.05, *** *p* ≤ 0.001 in comparison to negative controls, ≠≠ *p* ≤ 0.01 and ≠≠≠ *p* ≤ 0.001 in comparison to positive controls). PSPE: Polyphenolic fraction of *P. sativum*.

Figure 4 shows the variation in the time spent at the center of the open-field test during the three weeks of extract treatments. It can be seen that mice treated with PSPE (50 and 100 mg/kg) spent more time in the central area compared to the control groups. This significant increase is proportional not only to the duration of treatment but also to the concentration of the extract. The optimal value was obtained with a concentration of 100 mg/kg (37.4 s ± 1.778, compared to 33.4 s ± 1.208 sat a dose of 50 mg/kg). This indicates an anxiolytic effect of this plant.

**Figure 5:**
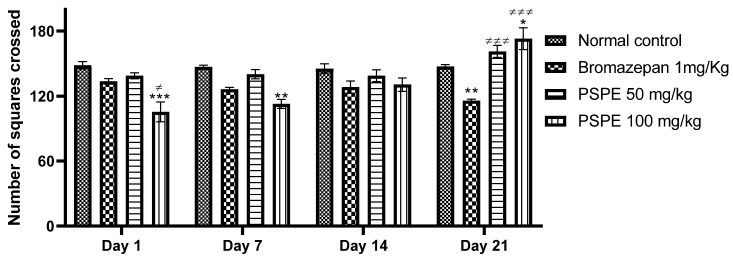
Variation in the number of tiles traversed during the open-field test over the four weeks of treatment in control and treated mice (* *p* ≤ 0.05, ** *p* ≤ 0.01, *** *p* ≤ 0.01 in comparison to negative controls, ≠ *p* ≤ 0.05, ≠≠≠ *p* ≤ 0.001 in comparison to positive controls). PSPE: Polyphenolic fraction of *P. sativum*.

The variation in the number of tiles crossed during the three-week experiment is shown in Figure 5. Analysis showed a remarkable increase in the number of tiles traversed by PSPE-treated mice (50 mg/kg (161.2 ± 5.490), 100 mg/kg (173 ± 10.104)) compared to the bromazepam 1 mg/kg (115.8 ± 1.393) and vehicle (147.4 ± 1.568)-treated groups. These results indicate a greater anxiolytic effect of FPPS even than that of bromazepam at 1 mg/kg.

#### 2.3.2. Anxious Behavior in the Light-Dark Room

**Figure 6:**
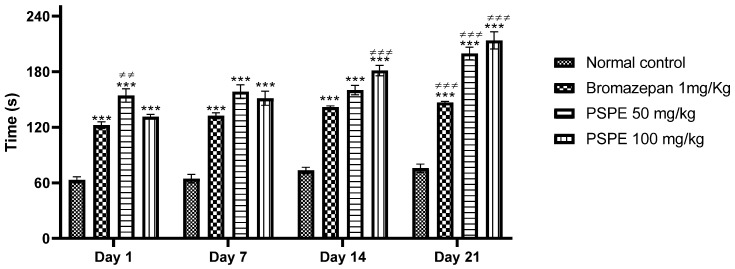
Variation in time spent in the lighted chamber during the three weeks of treatment in control and treated mice (*** *p* ≤ 0.001 in comparison to negative controls, ≠≠ *p* ≤ 0.01 and ≠≠≠ *p* ≤ 0.001 in comparison to positive controls). PSPE: Polyphenolic fraction of *P. sativum*.

Figure 6 presents the results of the variation in time spent in the lighted chamber during the study period. The results show a progressive increase in the time spent in the lighted chamber over time for mice treated with PSPE. This increase is especially noticeable on day 21. In this test, bromazepam-treated and PSPE-treated mice spent significantly more time in the lighted compartment (bromazepam 1 mg/kg (146.8 s ± 1.068), PSPE 50 mg/kg (199.6 s ± 6.838), PSPE 100 mg/kg (213.6 s ± 9.331) compared to control mice (vehicle (75.8 s ± 4.352)). This was construed as evidence of an anxiolytic effect of *P. sativum* polyphenolic extract.

**Figure 7:**
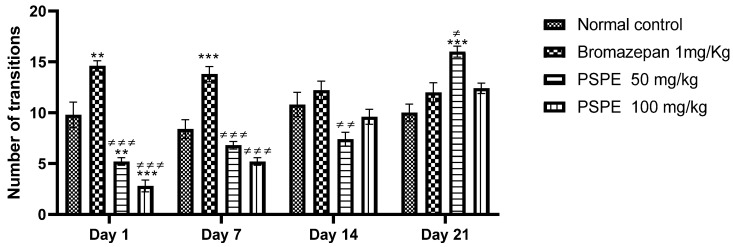
Variation in the number of transitions during the four weeks of treatment in control and treated mice (** *p* ≤ 0.01 and *** *p* ≤ 0.001 in comparison to negative controls, ≠ *p* ≤ 0.05, ≠≠ *p* ≤ 0.01 and ≠≠≠ *p* ≤ 0.001 in comparison to positive controls). PSPE: Polyphenolic fraction of *P. sativum*.

Figure 7 shows the variation in the number of transitions between the light and dark chambers during this test. A remarkable difference was found during the 21 days between PSPE-treated mice (50 mg/kg (16 ± 0.548), 100 mg/kg (12.4 ± 0.510)) and control mice (vehicle (10 ± 0.837), bromazepam 1 mg/kg (12 ± 0.949)). These results are consistent with the results of the time spent in the lighted chamber, as these two parameters are inversely proportional to the level of anxiety the mice displayed.

## 3. Discussion

We demonstrated in this study that the phenolic extract of *P. sativum* displays antioxidant activity in vitro and anxiolytic and antidepressant activities in vivo.

Ferulic acid and cinnamic acid have been found to have antidepressant effects in some studies [17,18], while quercetin and hydroxytyrosol demonstrated an approved anxiolytic effect [19,20]. Those compounds could be behind the observed effect.

The effectiveness of an antioxidant can be exerted in different forms, such as the scavenging of free radicals, the decomposition of free radicals, and also the chelation of metal ions [17]. This activity can be evaluated by FRAP and DPPH assays, the latter of which, due to its rapidity, is often used to screen molecules present in plant extracts [18]. Our results showed antioxidant activity in the phenolic extract of parsley. Previous work by Hinneburg et al. [21] showed that the aqueous extract of parsley exerted weak inhibitory activity of the DPPH radical, with an IC_50_ on the order of 12.0 ± 0.10 mg/mL (compared to the polyphenolic extract in our study, 0.184 μg/mL). Conversely, the chelating effect of this extract was more effective compared to other extracts used in the same study.

Recent studies also found a link between mood disorders and oxidative stress [22,23] and psychological stress [24,25], thus opening new avenues preventing and/or managing anxiety and depression regarding the potential application of antioxidants. Desrumaux et al. [26] noticed that vitamin E deficit in mice’s brains raised levels of key signs of oxidative stress and anxiety-provoking behaviors. Likewise, mice given vitamin C have recently revealed that this compound has an antidepressant role based on the tail suspension test results [27]. Polyphenols, including flavonoids and phenolic acids, are well-known for their potent antioxidant effects [28,29]. The usage of these secondary phytochemical metabolites as a way of avoiding and managing anxiety and depression may be a promising strategy [9]. A study carried out by Akinci et al. [30] demonstrated that parsley is successful in minimizing stress-induced gastric damage when taken orally by supporting the antioxidant defense system of cells, which is reflected in an increase in mean tissue glutathione levels (53.31 ± 9.50) and the activities of superoxide dismutase (15.18 ± 1.05) and catalase (16.68 ± 2.29).

The open-field experiment is used to evaluate an animal’s emotional condition. Animals who have been separated from their home cage and put in a different setting often demonstrate distress and anxiety by presenting changes in all or some of the parameters, like a decrease in ambulatory activity, exploration, and immobilization, but an increase in grooming behavior [31]. *P. sativum* extract at a dose of 100 mg/kg produced a very significant anxiolytic effect, as displayed by the significant increase in time spent at the center of the field and total ambulatory activity. This increase was greater than that seen in mice treated with bromazepam at 1 mg/kg.

The light-dark room experiment is also useful in predicting the anxiolytic effect of medications. In order to measure the degree of anxiety in mice during this test, two parameters are noted: (1) The lower percentage of time the animal spent in the dark chamber is related to its anxiety level. In other words, when the percentage of time spent in the lighted compartment is minimal, anxiety is deemed high, and (2) the number of transitions, i.e., the number of passages between the two compartments, is inversely related to the level of anxiety (lower number of transition means high anxiety level) [32]. The findings of this study revealed *P. sativum* polyphenolic extract at 100 mg/kg showed an optimal anxiolytic effect based on the animals’ increased time spent in the lighted chamber and the number of transitions compared with controls during the study period.

The forced swimming experiment was developed in the 1970s. It is also known by the name of its inventor as the Porsolt test [33]. Our finding indicates that the immobility time of the treated mice was shorter than that of the control mice, indicating an antidepressant effect of the phenolic extract of *P. sativum*. However, this decrease in the immobility time may have been in favor of either an increase in swimming time or climbing time. This difference is very important from a neuropharmacological point of view since theoretically, during forced swimming, antidepressants producing a predominantly noradrenergic or dopaminergic rise reduce immobility by increasing the time of escalation [34], whereas those that activate 5HT instead reduce immobility by increasing swimming time [35]. In terms of CNS function, the majority of polyphenols interact directly with neurotransmitter systems [36]. Studies conducted on parsley show a predominance of flavonoids [37]. The broad variety of flavonoids found in conventional traditional medicinal extracts are reported to have sedative/anxiolytic effects by direct binding to GABA A receptors [38].

A study carried out by Priprem et al. [39] indicates that the polyphenol quercetin demonstrates anxiolytic activity after a week of repeated administration at the dose of 300 mg/kg, which is not as effective as diazepam, which exhibits its effect one hour after administration at lower doses. Pereira et al. [40] showed that rosmarinic acid exhibits an anxiolytic effect at very low doses (2 to 4 mg/kg). Another example is apigenin, which demonstrates a selective and low affinity for benzodiazepine receptors producing an anxiolytic activity with almost no side effects [41]. Flavonoids also play a role in depression through inhibition of monoamine oxidase and the resulting increase in 5-HT, DA, and norepinephrine levels in certain areas of the brain [42].

Excitatory amino acids, such as aspartate, glutamate, homocysteine, and cysteine, stimulate post-synaptic cells, whereas inhibitory amino acids, such as alanine, glycine, GABA, and taurine, suppress post-synaptic cell development [43]. An enzyme called aromatic L-amino acid decarboxylase, also called DOPA decarboxylase, tryptophan decarboxylase, 5-hydroxytryptophan decarboxylase, or AAAD, which catalyzes the respective decarboxylation of l-dopa and 5-hydroxytryptophan into dopamine and 5-hydroxytryptamine may also play a role. Parsley may cause overexpression of this enzyme, contributing to its antidepressant effect. A study carried out on *Centella asiatica*, a plant belonging to the same Apiaceae family, showed that the antidepressant effect of total triterpenes was due to the improvement of the function of the hypothalamic-pituitary-adrenocortical axis and the increase in the content of monoamino neurotransmitters [44]. These studies suggest the presence of anxiolytic and antidepressant activity by the phenolic extract of *P. sativum*.

## 4. Materials and Methods

### 4.1. Plant Material

*Petroselinum sativum* Hoffm. aerial parts were collected during the pre-flowering phase (spring 2018) from Tanounate region in North Morocco (34°32′9″ N 4°38′24″ W). Classification, identification, and botanical name of this plant were verified by a qualified botanist Pr. Bari Amina. The plant’s sample is deposited at the Faculty of Sciences Dhar El Mahrez Fez herbarium (voucher specimen: 18TA5001).

### 4.2. Extraction

Extraction was carried out as described by Slighoua et al. [11]. Briefly, 10 g of the dried aerial parts of *P. sativum* fine powder was mixed with methanol (100 mL) and macerated for 3 h at a temperature of 50 °C. Afterwards, the filtrate was concentrated using a rotary evaporator until dryness to ensure complete solvent evaporation. The resulted extract was redissolved in distilled water (200 mL) and washed out with three hexane and chloroform (200 mL three times) to get rid of the pigments and other impurities. The final aqueous phase was extracted with ethyl acetate (200 mL three times). The organic phase (ethyl acetate) was concentrated to obtain the polyphenol extract. The yield of the extraction is 10.52%.

Our previously published work on the same extract demonstrated by using HPLC-DAD analysis that it is composed of the following polyphenols: (1) Ferulic acid, (2) Cinnamic acid, (3) Gallic acid, (4) Quercetin, (5) Myricetin, (6) Naringenin, (7) Hydroxytirosol [11].

### 4.3. Study Animals

In this study, mice (Swiss albino) were provided by the Animal House of the faculty of sciences Dhar el Mahraz Fez. Before being included in the experiments, they were put in groups of six in conventional cages and were allowed a two-week adaptation period with free access to food and water and a controlled temperature of 22 ± 2 °C and under a light/dark cycle of 12 h/12 h.

Experiments were performed pursuant to international standards for the Treatment and Use of Experimental Animals [16] and the Internal Animal Ethics Committee (#09-12/2019/LBEAS).

### 4.4. Evaluation of the Antioxidant Activity

#### 4.4.1. 2,2-Diphenyl-1-picrylhydrazyl (DPPH) Test

DPPH is one of the most common chemical compounds used for assessing the antioxidant activity of phenolic compounds [45]. DPPH possesses an unpaired electron on a bridge atom of nitrogen. The principle of this test is summarized in the capacity of the extract to reduce the dark purple DPPH free radical into a yellowish-colored reduction measurable by spectrophotometry [28]. A series of extract concentrations was prepared in methanol, 100 µL of each of which was added to 750 µL of a methanolic solution of DPPH (0.004%). After an incubation period of 30 min at 25 °C, the absorbance was measured at 517 nm. For the negative control, the sample was replaced by methanol. The percentage of DPPH was determined by the following equation:

I = [(Blank − Sample)/Blank] × 100(1)

I is the percentage of antiradical activity, Sample is the absorbance of the sample, and Blank is the absorbance of negative control.

#### 4.4.2. Ferrous Reducing Power (FRAP) Test

The reducing power of iron (Fe^3+^) in the extract studied was determined according to the method described by Mechchate et al. [28]. Briefly, 100 μL of the extract at different concentrations was mixed with 500 μL of a phosphate-buffered solution (PBS, 0.2 M, pH 6.6) and 500 μL of a 1% solution of potassium ferricyanide K_3_Fe(CN)_6_. These were incubated in a water bath at 50 °C for 20 min. Then, 500 μL of a 10% aqueous solution of trichloroacetic acid (TCA), 100 μL of a 0.1% solution of ferric chloride FeCl_3_, and 0.5 mL of distilled water were added to the reaction mixture. The absorbance reading of this mixture was taken at 700 nm against a blank containing all the reagents of the medium except the plant extract. The results are expressed as 50% effective concentration (EC50), which represents the concentration of antioxidants necessary to obtain an absorbance of 0.5. An increase in absorbance corresponds to an increase in the reducing power of the extract tested.

### 4.5. Antidepressant Activity

#### Forced Swimming Test (FST)

The Porsolt model, or forced swimming test, is a predictive test of antidepressant-type activity. Mice are individually forced to swim in a cylindrical container filled to the height of 12 cm with water. The test lasts 6 min, but only the last 4 min of the test are analyzed. The animal is considered immobile when it floats in an upright position and makes only a few movements in order to maintain its balance in the water (immobility time).

A total of four batches of five mice each were used for this test. All plant extracts were administered by gavage for 21 days, and the test was performed 1 h after the administration of the extracts on 21 days. Group A, the negative control, received a physiological solution as a vehicle. Group B, the positive control group, received paroxetine at 11 mg/kg. Groups C and D received PSPE at 50 and 100 mg/kg, respectively.

One hour after plant extract administration, the immobility time was counted for 6 min. The first two minutes allowed the mouse to adapt to the stress, and then, the mouse became tired and immobilized with time. The immobility time was counted in seconds, and then, it was compared with the immobility time of mice in the positive control group (paroxetine) for reference [33].

### 4.6. Anxiolytic Activity

#### 4.6.1. Open Field

The open-field (OF) test is used to predict anxiolytic activity, as animals show a high degree of avoidance of a central area relative to the periphery. It is also used as an exploration and locomotion test. The area consisted of four wooden walls 40 cm high arranged in an area of 50 × 50 cm, divided into 25 tiles of equal size marked by black lines.

A total of four batches of five mice each were used for this test. All plant extracts were administered by gavage for 21 days. Next, the test was performed 1 h after the plant extract administration on days 1, 7, 14, and Group A, the negative control, received a physiological solution as a vehicle Group B, the positive control, received bromazepam at 1 mg/kg. Groups C and D received PSPE at 50 and 100 mg/kg, respectively.

After 60 min, the test was performed by placing each mouse in the central square to explore the arena to measure the total ambulatory activity of the mice (the number of total tiles crossed by the mice, i.e., the number of peripheral tiles and the number of central tiles crossed by the mice with all four legs) and the time spent in the center of the open field [31,46].

#### 4.6.2. Light-Dark Room Test

The light-dark room test is also used to predict anxiolytic activity, as it allows easy evaluation of anxiety-related animal behaviors by analyzing the movements of the animal between two compartments of different color and illumination. More anxious animals tend to spend less time in the lit room. The wooden light-dark box with dimensions 44 × 21 × 21 cm consisted of two black and white compartments, connected by a passage (a hole of 7 × 7 cm in the center, separating the two compartments and allowing access to one or the other of the compartments). The illuminated compartment was illuminated by a lamp.

A total of four batches of five mice each were used for this test. All plant extracts were administered by gavage for 21 days. The test was performed 1 h after the plant extract administration on days 1, 7, 14, and 21. Group A, the negative control, received a physiological solution as a vehicle. Group B, the positive control, received bromazepam at 1 mg/kg. Groups C and D received PSPE at 50 and 100 mg/kg, respectively.

After 60 min, the five-minute test was performed by placing each mouse facing the opening joining the dark compartment [47]. After each test, the box was cleaned with alcohol. The parameters measured were the time spent in the lighted chamber and the number of chamber transitions [48].

## 5. Conclusions

Parsley, a daily used culinary herb worldwide, presents enormous health benefits and through this study, it has been shown to have a remarkable antidepressant-like and anxiolytic activity, even better than classic drugs, especially with the dose of 100 mg/kg. In the search for an effective medicine with fewer or almost no side effect, this plant could be a well-placed alternative. This work encourages its daily consumption as well as its development to a well-established phytomedicine.

Further research is needed to complete this work, including the performance of other behavioral tests with the same extract to confirm its efficacy. Thus, a bioguided fractionation of the phenolic extract of *P. sativum* is planned to identify the key component responsible for the observed anxiolytic and antidepressant-like activities and to determine their precise mechanism of action.

## Data Availability

Data are available upon reasonable request.

## Sample Availability

Samples of the compounds are available from the authors.
